# Challenges to peer support in low- and middle-income countries during COVID-19

**DOI:** 10.1186/s12992-020-00622-y

**Published:** 2020-09-25

**Authors:** Richard Mpango, Jasmine Kalha, Donat Shamba, Mary Ramesh, Fileuka Ngakongwa, Arti Kulkarni, Palak Korde, Juliet Nakku, Grace K. Ryan

**Affiliations:** 1 Research and Training Section, Butabika National Referral Hospital, Kampala, Uganda; 2 Mental Health Section, MRC/UVRI and LSHTM Uganda Research Unit, Entebbe, Uganda; 3 Mental Health Department, School of Health Sciences, Soroti University, Arapai, Uganda; 4 Centre for Mental Health Law and Policy, Pune, India; 5 Department of Health Systems, Impact Evaluation and Policy, Ifakara Health Institute, Dar es Salaam, Tanzania; 6 Department of Psychiatry and Mental Health, Muhimbili University of Health and Allied Sciences, Dar es Salaam, Tanzania; 7 Psychiatry, Butabika National Referral Hospital, Kampala, Uganda; 8 Department of Population Health, Faculty of Epidemiology and Population Health, London School of Hygiene and Tropical Medicine, London, UK

**Keywords:** Peer support, Global mental health, Covid-19

## Abstract

**Background:**

A recent editorial urged those working in global mental health to “change the conversation” on coronavirus disease (Covid-19) by putting more focus on the needs of people with severe mental health conditions. UPSIDES (**Using Peer Support In Developing Empowering mental health Services**) is a six-country consortium carrying out implementation research on peer support for people with severe mental health conditions in high- (Germany, Israel), lower middle- (India) and low-income (Tanzania, Uganda) settings. This commentary briefly outlines some of the key challenges faced by UPSIDES sites in low- and middle-income countries as a result of Covid-19, sharing early lessons that may also apply to other services seeking to address the needs of people with severe mental health conditions in similar contexts.

**Challenges and lessons learned:**

The key take-away from experiences in India, Tanzania and Uganda is that inequalities in terms of access to mobile technologies, as well as to secure employment and benefits, put peer support workers in particularly vulnerable situations precisely when they and their peers are also at their most isolated. Establishing more resilient peer support services requires attention to the already precarious situation of people with severe mental health conditions in low-resource settings, even before a crisis like Covid-19 occurs. While it is essential to maintain contact with peer support workers and peers to whatever extent is possible remotely, alternatives to face-to-face delivery of psychosocial interventions are not always straightforward to implement and can make it more difficult to observe individuals’ reactions, talk about emotional issues and offer appropriate support.

**Conclusions:**

In environments where mental health care was already heavily medicalized and mostly limited to medications issued by psychiatric institutions, Covid-19 threatens burgeoning efforts to pursue a more holistic and person-centered model of care for people with severe mental health conditions. As countries emerge from lockdown, those working in global mental health will need to redouble their efforts not only to make up for lost time and help individuals cope with the added stressors of Covid-19 in their communities, but also to regain lost ground in mental health care reform and in broader conversations about mental health in low-resource settings.

## Background

A recent editorial published in *Lancet Psychiatry* urged mental health professionals to “change the conversation” on mental health and coronavirus disease (Covid-19) by putting more focus on the needs of people with severe mental health conditions [1]. The editorial praised a “few honourable exceptions” in which media outlets had actively reported on issues relevant to people with severe mental health conditions during the Covid-19 pandemic; however, these examples were entirely from high-income countries. The authors noted that even prior to the outbreak, the discourse in global mental health has been dominated by common mental health conditions, for which interventions are perhaps seen as more easily delivered and scalable in low- and middle-income country (LMIC) settings. Indeed, Misra et al.’s (2019) systematic review of the scientific literature using the term “global mental health” identified more than twice as many empirical studies on depression (*n* = 33) compared to psychosis (*n* = 14) in the years 2007–2016 [2]. These trends will continue if more attention is not paid to people with severe mental health conditions in LMICs when considering the effects of the Covid-19 pandemic on individuals, communities and services during this challenging time.

UPSIDES (**Using Peer Support In Developing Empowering mental health Services**) is a six-country consortium carrying out implementation research on peer support for people with severe mental health conditions in a range of high- (Germany, Israel), lower middle- (India) and low-income (Tanzania, Uganda) settings [3–5]. As with many other studies of interventions that rely heavily on face-to-face contact, UPSIDES’ multi-site randomized controlled trial was put on pause following the outbreak, just a few months after participant recruitment started in January 2020. This happened at a precarious early stage of implementation, when many newly trained peer support workers (PSWs) were just starting to develop the mutually supportive relationships with their peers and with each other that are the mainstays of peer support interventions [6]. The purpose of this commentary is to briefly outline some of the key challenges faced by our LMIC partners as a result of Covid-19, and to share some early lessons learned that might also apply to other services seeking to address the needs of people with severe mental health conditions in similar contexts.

### Challenges in low-resource settings

In Uganda, where peer support has been available since 2011, the country’s “total lockdown” resulted in a ban on public and private transport, severely restricted outpatient mental health care and closure of rehabilitative and other essential services. As in many countries around the world [7], Uganda’s PSWs are not salaried employees and instead combine small incentives such as meal and transport allowances with income-generating activities such as farming or petty-trading to make ends meet. Lockdown has put their livelihoods under threat, while also restricting access to the services upon which many PSWs rely to support their own recovery. Meanwhile, PSWs have been unable to meet fellow PSWs or the peers whom they support and who are often facing similar challenges, contributing to loneliness and isolation—and ultimately to the deterioration of some PSWs’ mental health. While most PSWs have mobile phones, many of their peers do not, and paying for cellular service and data can be a hardship.

Similar challenges have been observed at UPSIDES study sites in India and Tanzania, where many do not have their own phones and outpatient mental health care has been temporarily restricted. The study site in India recruits its PSWs (called “peer support volunteers”, or “PSVs”) through the Department of Health and Family Welfare as part of the Government of Gujarat’s mental health policy, putting PSVs in a less financially precarious situation. However, this also means that peer support is treated like other hospital-based services and does not extend into the community. The PSV intervention, which was initiated in 2015 as part of the QualityRights Gujarat programme, has never included home visits, and PSVs do not always feel they have developed close enough relationships with their peers to carry their conversations forward by phone. When the lockdown was announced in late March, PSVs were asked not to come to the hospital, for their own safety—effectively cutting off PSVs from their peers. In Tanzania, the government has not imposed strict lockdown measures, but many remain fearful of Covid-19. Some PSWs’ attempts to continue providing face-to-face support through home visits are no longer welcomed by peers, necessitating a pause to the trial. Unlike at other study sites, the peer support intervention is new to Tanzania, and PSWs are paid on a per-activity basis using three-month research contracts that have proven difficult to renew while the trial is stopped.

At each site, efforts are being made to maintain weekly contact with PSWs, with mixed results. In Uganda, nine of 15 PSWs have been able to participate in weekly conference calls, and an occupational therapist from the study team has begun carrying out home deliveries of PSWs’ and peers’ prescriptions by motorbike. In India, the research team has been able to stay in touch with three PSVs who have their own phones. The other seven have been contacted through their care providers, though this indirect contact makes it more difficult to provide social and emotional support to the PSVs. Nevertheless, the study team has been able to provide factual information about Covid-19 and safety measures, to help dispel harmful or distressing misinformation, and also to share self-care videos and other resources on mental health during the pandemic—largely via WhatsApp. In Tanzania, the research team reaches out to each PSW by phone, and while many of these PSWs share phones with other family members and are not always reachable, all have been contacted at least once.

### Lessons learned

The key take-away from these experiences to-date is that inequalities in terms of access to mobile phones, service and data, as well as to secure employment and/or benefits, put PSWs in LMICs in particularly vulnerable situations precisely when they and their peers are also at their most isolated. So, what recommendations can we make at this stage to help establish more resilient peer support interventions in low-resource settings?

First, recognize from the outset that people with severe mental health conditions in LMICs are often in precarious situations when disaster strikes, and prepare accordingly. Only 14% of low-income countries and 38% of lower middle-income countries provide government benefits to the majority of people with severe mental health conditions, and these benefits are often insufficient to meet basic needs [8]. This makes insecure contracts for PSWs especially problematic when there is any interruption to a peer support service, and PSWs may be unable to afford to stay in contact with either their peers or the service. In the long-term, we would advocate for more public-funded, paid peer positions within mental health services in LMICs, extending to PSWs the same employee benefits available to the clinicians whom they work alongside. In the short- to medium-term, we would advise projects that work with PSWs in LMICs to abandon hourly or pay-per-activity models of reimbursement and to offer health insurance and other employee benefits where possible, to better protect PSWs. These benefits should ideally include mobile phones with Internet access and a basic allowance for phone credit and mobile data. Second, remember that people with severe mental health conditions are among those most often left behind from disaster response efforts, as argued by Michelle Funk and colleagues (2010) at the World Health Organization (WHO) [9]. A peer support service may need to expand its scope of work to ensure that PSWs and peers have access to the basic information and supplies that they need to stay well in these extreme circumstances, as has been the case in India and Uganda. Unfortunately, while food, medications and other essentials can be delivered by motorbike or some other form of personal transport, the alternatives for delivering psychosocial interventions are less straight-forward. One good that could perhaps emerge from Covid-19 is increased attention to the mental health and well-being of care providers [10] and the need for scalable interventions, such as the WHO’s Self-Help Plus, for emergency situations [11]. Adapting these resources for use by PSWs and peers could prove beneficial both in terms of responding to Covid-19 and as part of contingency-planning in LMICs, which are disproportionately affected by natural disasters and conflict [12].

This brings us to our third and final recommendation: acknowledge that while it is extremely important to remain in regular, direct contact with PSWs and peers, there is no perfect substitute for face-to-face interaction. Across all UPSIDES sites, including in high-income countries, teams lament the impersonality of technological “solutions” that make it more difficult to observe individuals’ reactions, talk about emotional issues and offer appropriate support— support that is just as important for PSWs as it is for peers [13]. While there have undoubtedly been some positive experiences of remote PSW in response to Covid-19, particularly in high-income countries [14], the barriers to access in low-resource environments compound the inherent challenges of maintaining a human connection online or by phone.

## Conclusions

In summary, improving access to mobile phones, cellular service and data, providing appropriate insurance and employee benefits for PSWs, and working proactively to address the contracting issues that leave PSWs vulnerable, can all help to better protect PSWs in LMICs. Further, momentum in the development of scalable psychosocial interventions should be harnessed to develop more holistic packages of support for emergency situations. However, these are only a few early lessons from UPSIDES, and we expect to encounter much harsher realities of Covid-19’s impact on people with severe mental health conditions in LMICs as restrictions lift and PSWs and peers begin returning to services.

In environments where mental health care was already heavily medicalized and mostly limited to medications issued by psychiatric institutions, Covid-19 threatens burgeoning efforts to pursue a more holistics and person-centered model of care for people with severe mental health conditions. We cannot assume that the supports on which people rely for their recovery can simply be suspended or substituted with phone calls without any negative ramifications; neither can we afford to go backwards. As countries emerge from lockdown, we will need to redouble our efforts not only to make up for lost time and help individuals cope with the added stressors of Covid-19 in their communities, but also to regain lost ground in mental health care reform and in broader conversations about global mental health in LMICs.

## Data Availability

Not applicable.

## Abbreviations

Covid-19: Corona virus disease 2019
LMICs: Low- and middle-income countries
PSV: Peer support volunteer
PSW: Peer support worker
UPSIDES: Using Peer Support In Developing Empowering mental health Services
